# Transcatheter Valve-in-Valve and Iatrogenic ASD Closure for Tricuspid Thrombosis in Heparin-Induced Thrombocytopenia

**DOI:** 10.1016/j.jaccas.2026.106870

**Published:** 2026-02-18

**Authors:** Waseem Raja, Yixin Zhang, Darin Busby, Georges El Khoury, Ali Zgheib, Hussam F. Hawamdeh, Imraan Ansaarie, Valentin Suma, Thomas Zeyl, Daniel E. Soffer, Erol Belli, Calvin Choi

**Affiliations:** a University of Florida College of Medicine–Jacksonville, Jacksonville, Florida, USA; b Advanced Endovascular Institute, St Augustine, Florida, USA

**Keywords:** bioprosthetic valve thrombosis, hypercoagulable state, prosthetic valve dysfunction, transcatheter cardiac intervention, transesophageal echocardiography

## Abstract

**Background:**

Early bioprosthetic valve thrombosis is a life-threatening complication, and hypercoagulable states such as heparin-induced thrombocytopenia (HIT) can contribute to the formation of thrombi.

**Case Summary:**

A 67-year-old woman with a prior history of HIT and surgical replacement of mitral and tricuspid valves presented with shortness of breath. Transthoracic echocardiography showed an atrial septal defect with right-to-left shunt and thickened tricuspid valve leaflets. Intraoperative transesophageal echocardiography revealed a severe thrombotic bioprosthetic tricuspid valve. She underwent successful transcatheter tricuspid valve-in-valve (TTViV) and atrial septic device closure.

**Discussion:**

TTViV is a critical option for patients presenting with early tricuspid valve thrombosis due to HIT. Increased right atrial pressure from tricuspid thrombosis can result in dehiscence of the interatrial sutures, leading to a hemodynamically significant shunt and necessitating its closure.

**Take-Home Messages:**

HIT is a prothrombotic state that can lead to early valve thrombosis. TTViV offers an effective treatment option for high–surgical risk patients.

## Visual Summary

**Figure:**
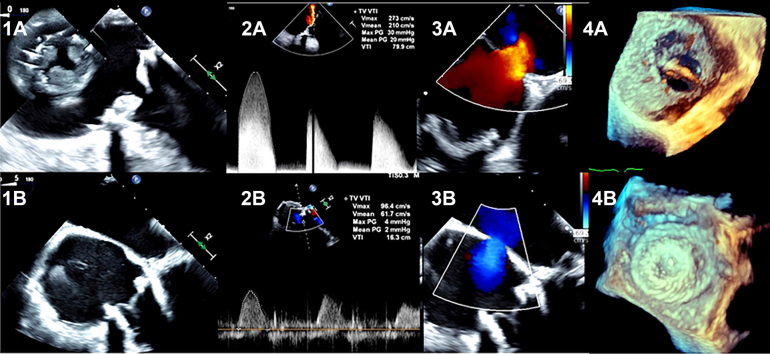

## History of Presentation

A 67-year-old woman was known to have severe mitral stenosis and mitral regurgitation, likely rheumatic in origin, and heparin-induced thrombocytopenia (HIT). Her HIT rapid antibody test was positive. HIT 4T score was 5 (intermediate range), and unfractionated heparin serotonin release assay was negative. She had undergone surgical mitral valve replacement via median sternotomy approach with a 31-mm Epic bioprosthetic valve, tricuspid valve replacement with a 31-mm Epic bioprosthetic valve, maze atrial ablation, and ligation of the left atrial appendage with a 40-mm AtriClip. The transseptal approach was used for the mitral valve replacement, and the atriotomy site was closed with Prolene sutures in 2-layer fashion, although no patch was used. Given the high risk of the index surgical procedure, she received unfractionated heparin only during the initial phase of the bypass, and postoperatively, bridging was performed with intravenous argatroban. Her platelet count had remained normal during the hospital stay. Her immediate postoperative course had been uneventful, and she was discharged on warfarin treatment.

Six months after her initial surgery, she presented with shortness of breath that had started approximately 1 week prior. On arrival, she was hypoxic with an oxygen saturation of 80% on high-flow oxygen therapy, pulse of 68 beats/min, and blood pressure of 136/86 mm Hg. She required a nonrebreather mask to improve her oxygen saturation to >90% and subsequently received noninvasive ventilation, with FiO_2_ of 0.80. The international normalized ratio was 1.18, as she had developed a hemothorax before this admission and had a period of coumadin discontinuation. D-dimer was 3.34 μg/mL (normal range: <0.50 μg/mL). She underwent work-up for possible pulmonary embolism, transthoracic echocardiography (TTE), and swabs for viral infections.

## Past Medical History

The patient's medical history included HIT, heart failure with reduced ejection fraction, and paroxysmal atrial fibrillation.

## Differential Diagnosis

Considerations included pulmonary embolism, hemodynamically significant atrial septal defect (ASD), tricuspid bioprosthetic valve thrombosis, and tricuspid bioprosthetic valve stenosis.

## Investigations

**Figure 1:**
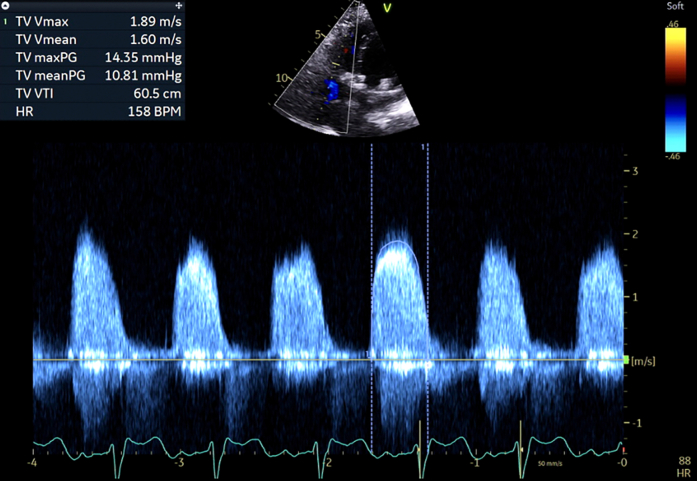
Preprocedural TTE TTE showing a gradient of almost 11 mm Hg across the tricuspid valve. TTE = transthoracic echocardiography.

The patient's swabs were negative for respiratory syncytial virus, COVID, and flu. Computed tomography (CT) was negative for pulmonary embolism. TTE (Figure 1) revealed global systolic dysfunction with an ejection fraction of 35%. The mitral valve had a 31-mm Epic bioprosthetic valve well seated, with visible struts, mobile leaflets, and a mean transmitral gradient of 6.6 mm Hg. The tricuspid position revealed a bioprosthetic valve with restricted leaflets and a mean gradient of 10.8 mm Hg, consistent with moderate tricuspid stenosis. There was a tissue dropout of 1.1 cm diameter at the middle of the interatrial septum, with a right-to-left shunt and immediate crossover of bubbles from right to left on the agitated bubble study.

## Management/Intervention

**Figure 2:**
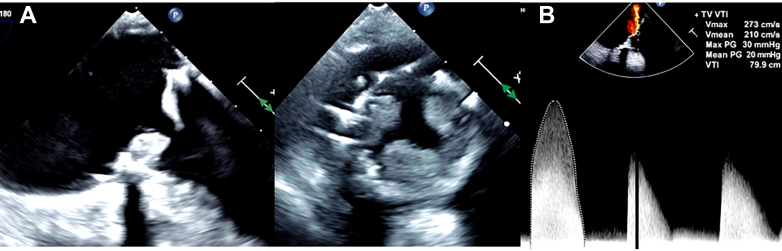
Intraoperative TEE (A) TEE midesophageal 4-chamber view showing severely thickened bioprosthetic tricuspid valves in the setting of thrombosis. (B) Continuous-wave Doppler across the tricuspid valve demonstrating severe obstruction before intervention (mean gradient: 20 mm Hg at a heart rate of 87 beats/min). TEE = transesophageal echocardiography.

Owing to persistent hypoxia despite bilevel positive airway pressure support in the upright position, the patient's case was discussed in a heart team meeting, and she was found to be at very high surgical risk given her recent sternotomy and history of HIT. We elected to proceed with emergent transcatheter intervention. Intraoperative transesophageal echocardiography (TEE) (Figures 2A and 2B, Video 1) revealed a severely thickened tricuspid valve; leaflets appeared significantly thickened on the ventricular side, with markedly restricted motion on both two- and three-dimensional imaging. The peak velocity was 2.73 m/s, and the mean transvalvular gradient was 20 mm Hg at a heart rate of 87 beats/min. These findings were highly suggestive of prosthetic valve (PV) thrombosis. Color Doppler confirmed interatrial septal defect with right-to-left shunting. Tricuspid thrombosis, leading to elevated right atrial pressures, likely contributed to the dehiscence of the closed suture site, resulting in a significant shunt. In a transseptal approach for mitral surgery, a patch is typically used if there is an existing ASD or patent foramen ovale. However, in this case, only suture closure was performed because there was no existing shunt. Swan-Ganz catheter showed a mean pulmonary artery pressure of 29 mm Hg. It was an unfavorable condition to close the shunt with right-to-left shunt.

**Figure 3:**
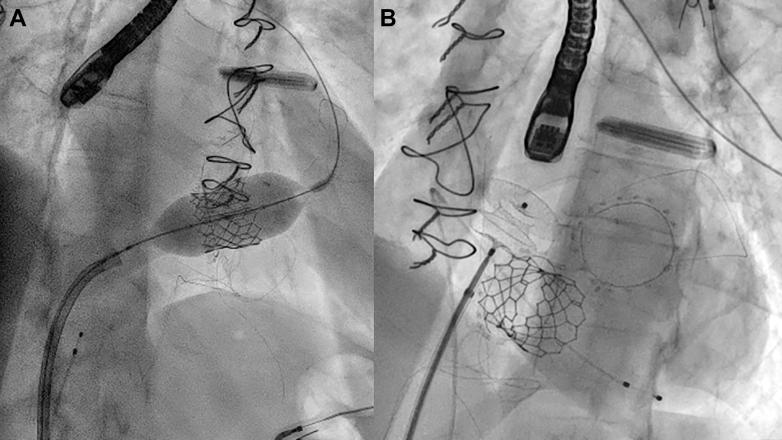
Intraprocedural Fluoroscopic Images (A) TTViV using a 29-mm Sapien S3 valve. (B) Closure of the ASD with an 18-mm Amplatzer device. ASD = atrial septal defect; TTViV = transcatheter tricuspid valve-in-valve.

We decided to proceed with an emergent transcatheter tricuspid valve-in-valve (TTViV) procedure. The previously deployed 31-mm Epic bioprosthetic valve has a stent internal diameter of 29 mm and true internal diameter of 27 mm; therefore, we decided to deploy a 29-mm Sapien S3 Resilia valve (Edwards Lifesciences), although a formal CT scan could not be obtained. The Sapien S3 device was mounted on its delivery system and advanced via the right femoral vein sheath, positioning it across the bioprosthetic tricuspid valve. The valve was deployed under TEE and fluoroscopic guidance (Figure 3A, Video 2), with excellent position. We observed an immediate improvement in hemodynamics, oxygen saturation, and reversal of shunt flow from left to right. The patient was systemically anticoagulated with a bolus of bivalirudin, followed by an intravenous infusion, on the background of HIT.

**Figure 4:**
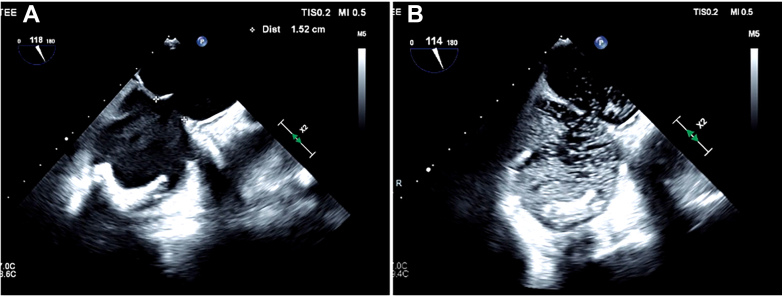
Intraprocedural TEE Assessment of the Septal Defect (A) ASD measuring 1.52 mm on TEE. (B) Agitated saline study after TTViV showed a bidirectional shunt across the ASD. ASD = atrial septal defect; TEE = transesophageal echocardiography; TTViV = transcatheter tricuspid valve-in-valve.

The intraprocedural TEE showed an ASD measuring 1.52 mm in the maximum dimension (Figure 4A). We then performed a bubble study, which revealed bidirectional flow predominantly in the left-to-right direction (Figure 4B, Video 3). The ASD was closed (Figure 3B, Video 4, Video 5, Video 6) with an 18-mm Amplatzer septal occluder (Abbott), resulting in complete resolution of the interatrial shunt. The patient's oxygen requirement decreased immediately, and we were able to extubate her at the end of the procedure with 100% oxygen saturation on room air.

## Outcome and Follow-Up

The patient was maintained on argatroban infusion postprocedure, with bridging to warfarin at discharge. She was stable and saturating at 98% on room air after the procedure and was discharged home in stable condition. On her 6-month follow-up visit, she was clinically stable, and TTE showed a well-seated tricuspid PV with no tricuspid regurgitation or paravalvular leak, peak velocity of 1.5 m/s, and mean gradient of 5.5 mm Hg. She will be kept on long-term warfarin anticoagulation.

## Discussion

TTViV can be a critical option for high–surgical risk patients presenting with early tricuspid valve thrombosis in an acute situation. An iatrogenic septal defect can become hemodynamically significant owing to high valve gradients, necessitating device closure, as shown in this case.

Thrombosis of bioprosthetic valves is a rare occurrence compared with thrombosis of mechanical valves, but it is more frequent on the right side of the heart because of a low-flow state. Observational studies show that the rate of symptomatic or hemodynamically significant bioprosthetic valve thrombosis is 0.3% in the first 30 days after valve implantation, and the long-term rate is <0.05% per year.^1^ HIT is a prothrombotic state in which platelet factor 4 antibodies are thought to activate platelets through FcγRIIa receptors, leading to drastic platelet activation. Activated platelets, endothelium, and leukocytes, together with derived microparticles, can lead to thrombin generation and coagulation. HIT has been reported to cause both native^2^ and prosthetic^3^ aortic valve thrombosis. A review of the broader literature shows that HIT can cause thrombosis at atypical sites, including cardiac valves, but we could not find a case of bioprosthetic tricuspid valve thrombosis possibly linked to HIT.

Patients with PV thrombosis can present with progressive dyspnea, signs of heart failure, or systemic embolization. It can be suspected in patients with acute or subacute dyspnea, particularly those with an increase in transprosthetic gradient. Right-sided thromboembolism may arise from pulmonary or tricuspid valve thrombus or vegetation. It can cause pulmonary embolism or paradoxical systemic embolism in patients with septal defects, patent foramen ovale, or other right-to-left shunts. The first-line imaging test is TTE, but acoustic shadowing caused by the prosthesis may limit visualization of thrombus, vegetations, and pannus. A combination of imaging and clinical criteria should be used to differentiate between PV thrombosis versus fibrotic pannus growth. Independent predictors of PV thrombosis include: increased transvalvular gradient (aortic P_max_: ≥50 mm Hg, mitral P_mean_: ≥10 mm Hg), presence of an occlusive mobile mass on the PV, and an international normalized ratio of ≤2.5.^4^ In patients with inconclusive TTE and TEE findings, multidetector CT can be considered. In our case, the patient was too unwell for these investigations, so only TEE was planned for intraoperative use.

## Conclusions

Our case report highlights important etiological and management aspects of early tricuspid valve thrombosis. HIT, being a hypercoagulable state, can lead to early valve thrombosis. TTViV can be a crucial management option for acutely unwell patients who are not surgical candidates, and alternative anticoagulants can be safely used for this procedure in patients with HIT. Although the large ASD in our patient contributed to hypoxemia, it also served as a vital compensatory mechanism by offloading elevated right atrial pressure. This case highlights the etiological association between HIT and PV thrombosis, emphasizing the importance of detailed imaging and hemodynamic assessment before septal closure. Addressing the underlying PV thrombosis must precede shunt elimination.

**VISUAL SUMMARY:**
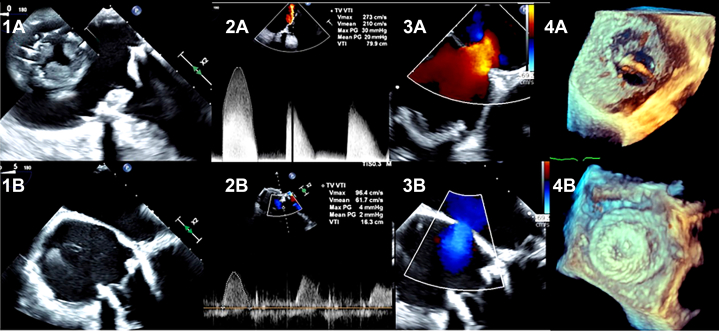
Transesophageal Echocardiography and Doppler Findings (1A) Prosthetic tricuspid valve thrombosis with severe stenosis and (1B) improved leaflet motion after valve-in-valve replacement. (2A) Preintervention continuous-wave Doppler across the tricuspid valve demonstrating severe obstruction (mean gradient: 20 mm Hg) and (2B) normalization postintervention (mean gradient: 2 mm Hg). (3A) Color Doppler showing right-to-left atrial shunt before tricuspid valve replacement and (3B) shunt reversal after valve replacement. (4A) Three-dimensional reconstruction of the interatrial septum before closure and (4B) after deployment of an Amplatzer septal occluder.

## Funding Support and Author Disclosures

The authors have reported that they have no relationships relevant to the contents of this paper to disclose.

**Take-Home Messages**

• Heparin-induced thrombocytopenia is a prothrombotic state that can lead to early valve thrombosis, particularly in low-flow valves such as the tricuspid valve.
• TTViV offers a safe and effective treatment option for high–surgical risk patients who are acutely unwell.
• Iatrogenic ASD can become hemodynamically significant given tricuspid valve thrombosis, leading to raised right atrial pressure and dehiscence of the sutures.

## Appendix

Video 1 TEE Midesophageal 4-Chamber View This view shows severely thickened bioprosthetic tricuspid valves in the setting of thrombosis. TEE = transesophageal echocardiography.

Video 2 Intraprocedural Fluoroscopic Images Post-TTViV and deployment of the 18-mm Amplatzer septal occluder device. TTViV = transcatheter tricuspid valve-in-valve.

Video 3 Agitated Saline Study After TTViV Showing Bidirectional Shunt Across the ASD ASD = atrial septal defect; TTViV = transcatheter tricuspid valve-in-valve.

Video 4 TEE Showing 2D Color Doppler After Deployment of the 18-mm Amplatzer Septal Occlude Device 2D = two-dimensional; TEE = transesophageal echocardiography.

Video 5 TEE 3D View of the 18-mm Amplatzer ASD Closure Device 3D = three-dimensional; ASD = atrial septal defect; TEE = Transesophageal echocardiography.

Video 6 Final Fluoroscopic Images After TTViV and Amplatzer Septal Occluder Deployment TTViV = transcatheter tricuspid valve-in-valve.

## References

[1] Mirsadraee S., Williams M.C., Mordi I. (2021). Bioprosthetic valve thrombosis and degeneration following transcatheter valve implantation. Clin Radiol.

[2] Guinn N., Tanaka K., Erdoes G. (2023). The year in coagulation and transfusion: selected highlights from 2022. J Cardiothorac Vasc Anesth.

[3] Faucher L., Marchandot B., Carmona A., Ohana M., Trimaille A., Morel O. (2023). Bioprosthetic valve thrombosis after transcatheter aortic valve replacement and pulmonary embolism due to heparin-induced thrombocytopenia: a case report. Front Cardiovasc Med.

[4] Budde R.P.J., Faure M.E., Abbara S. (2025). Cardiac computed tomography for prosthetic heart valve assessment. An expert consensus document of the Society of Cardiovascular Computed Tomography (SCCT), the American College of Cardiology (ACC), the European Society of Cardiovascular Radiology (ESCR), the North American Society of Cardiovascular Imaging (NASCI), the Radiological Society of North America (RSNA), the Society for Cardiovascular Angiography & Interventions (SCAI) and Society of Thoracic Surgeons (STS). J Cardiovasc Comput Tomogr.

